# Supplementary material for: Ring synthetic chromosome V SCRaMbLE
Source: Nat Commun. 2018 Sep 17;9:3783. doi: 10.1038/s41467-018-06216-y (PMC6141504; doi:10.1038/s41467-018-06216-y)
Supplement: Supplementary file 1 — Supplementary Information [file 41467_2018_6216_MOESM1_ESM.pdf]

Supplementary Material for
Ring Synthetic Chromosome V SCRaMbLE

Wang *et al.*

This PDF file includes:

Supplementary Figures 1 to 4

Supplementary Data 1 to 4

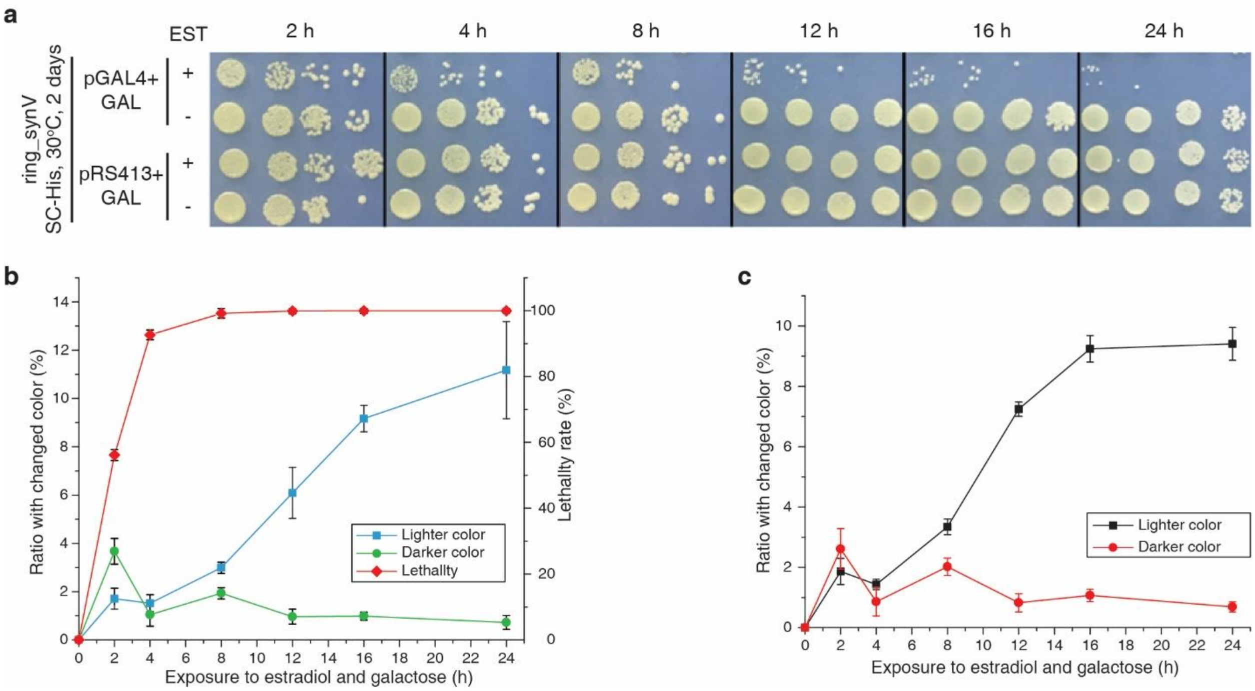

Supplementary Figure 1. Conditional chromosome evolution by SCRaMbLE.

a Ten-fold serial dilution assays of cells carrying ring_synV exhibited chromosome rearrangement and growth defects when inducted to galactose and estradiol. The patches of colonies from left to right were all serially diluted in 10-fold increments in water and plated onto SC–His media and incubated at 30°C for 2 days. GAL: galactose; EST: estradiol; Hours: exposure to estradiol. pGAL4 plasmid was used to expressing Cre.

b Lethality curve and rate of cells with changed phenotype for ring_synV strain during Cre induction time course.

c Rate of cells with changed phenotype for synV strain during Cre induction time course. Values are averages from three experiments, and error bars denote s.d..

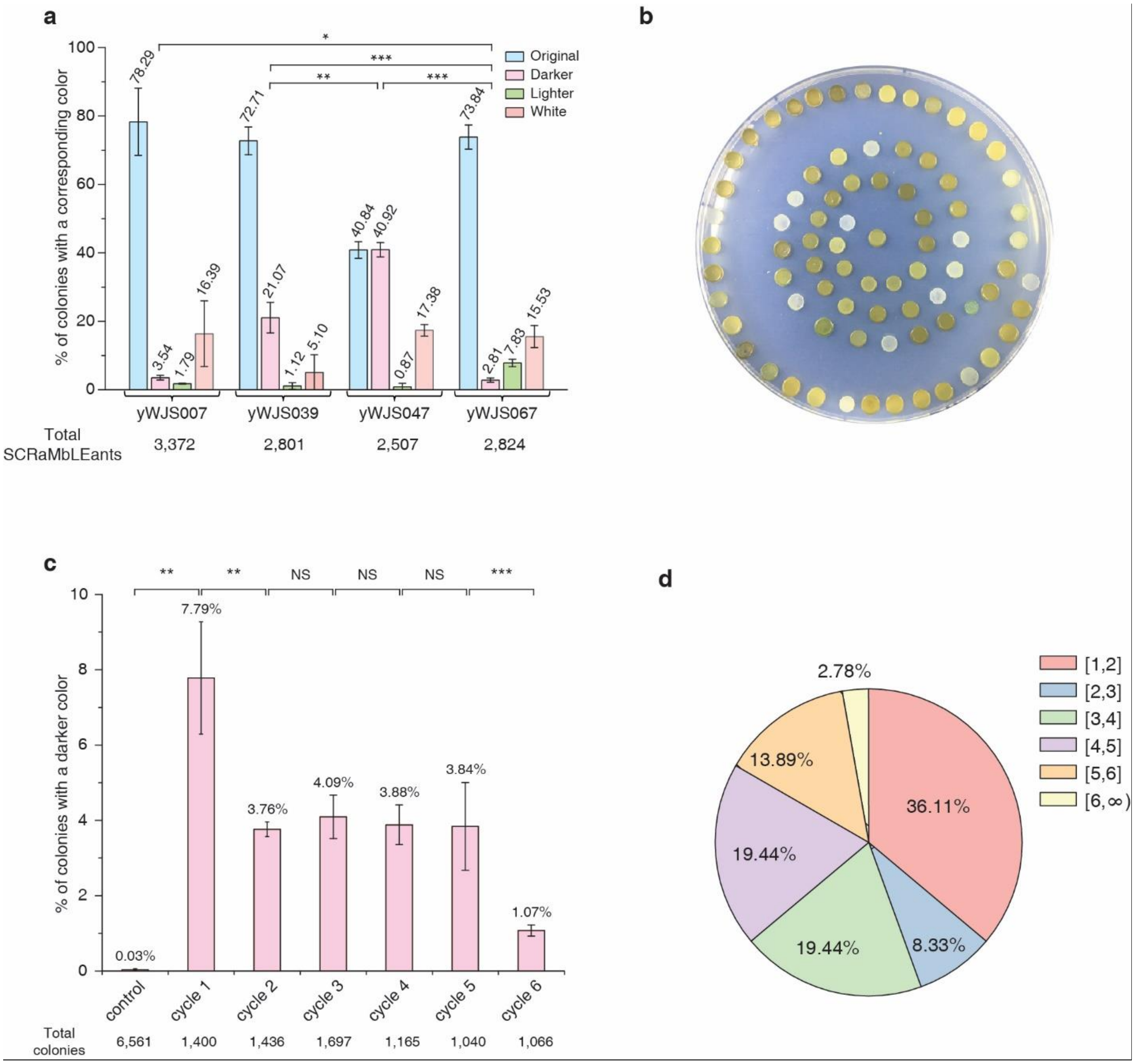

Supplementary Figure 2. Phenotypic diversity of SCRaMbLEants.

a Distribution of colonies with different phenotypes in the second cycle of SCRaMbLE.

b Phenotypic diversity of SCRaMbLEd ring_synV strain.

c Percent of colonies with a deepened color after the SCRaMbLE in each cycle.

d Fold changes of PDV production after each SCRaMbLE cycle, based on the total number of variable SCRaMbLEd colonies. Values are averages from three experiments, and error bars denote s.d.. T-tests measure significance of difference in colonies with darker colors. NS, not significant; * P<0.05; ** P<0.005; *** P<0.0001. See also Supplementary Data 2.

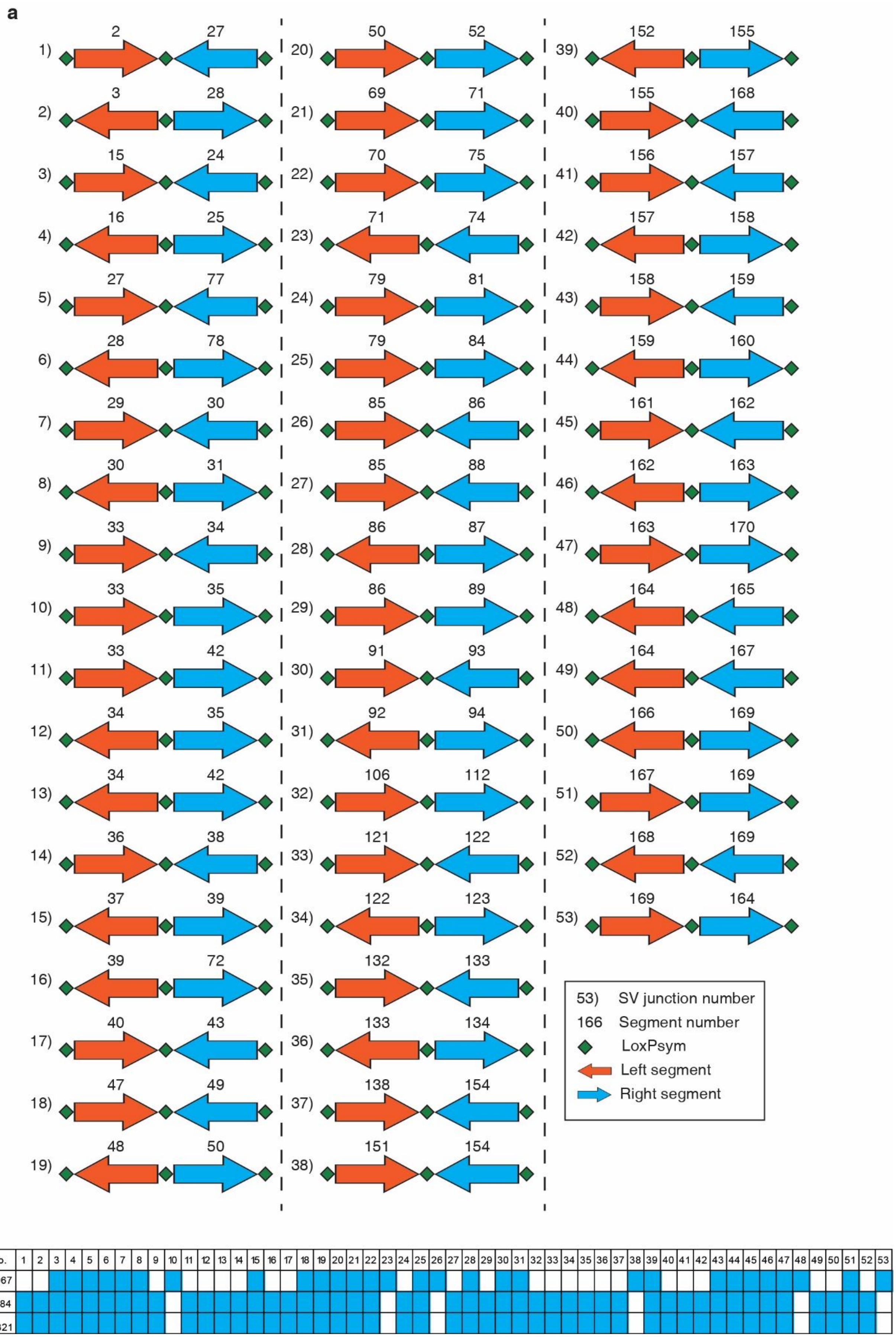
a
1) 2 27
2) 3 28
3) 15 24
4) 16 25
5) 27 77
6) 28 78
7) 29 30
8) 30 31
9) 33 34
10) 33 35
11) 33 42
12) 34 35
13) 34 42
14) 36 38
15) 37 39
16) 39 72
17) 40 43
18) 47 49
19) 48 50
20) 50 52
21) 69 71
22) 70 75
23) 71 74
24) 79 81
25) 79 84
26) 85 86
27) 85 88
28) 86 87
29) 86 89
30) 91 93
31) 92 94
32) 106 112
33) 121 122
34) 122 123
35) 132 133
36) 133 134
37) 138 154
38) 151 154
39) 152 155
40) 155 168
41) 156 157
42) 157 158
43) 158 159
44) 159 160
45) 161 162
46) 162 163
47) 163 170
48) 164 165
49) 164 167
50) 166 169
51) 167 169
52) 168 169
53) 169 164
53) SV junction number
166 Segment number
LoxPsym
Left segment
Right segment
b
SV No.
yWJS067
yWJS184
yWJS321

Supplementary Figure 3. Novel structural junctions revealed by WGS.

a Novel structural junctions detected by WGS in yWJS067, yWJS184 and yWJS321.

b Map of novel structural junctions in yWJS067, yWJS184 and yWJS321.

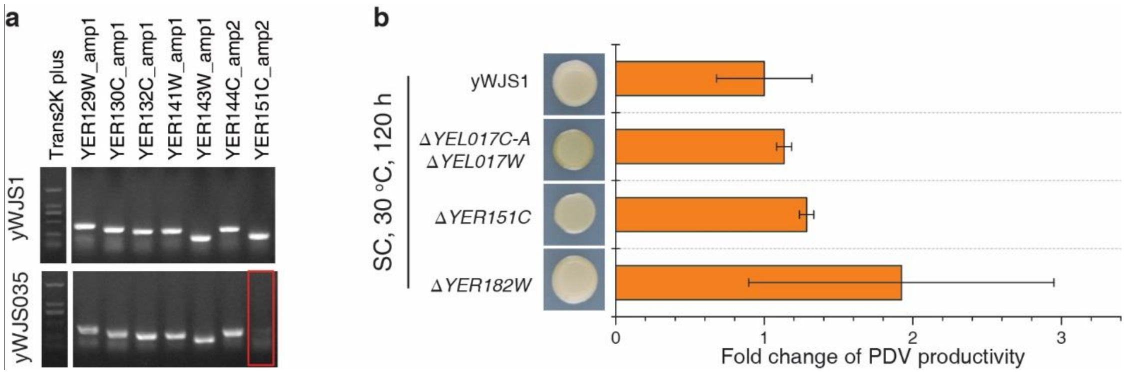

Supplementary Figure 4. Gene deletion led to improvement of PDV production.

a The PCRTagging analysis of the SCRaMbLEants with increased PDV biosynthesis revealed loss of genes. The red rectangle stands for the absence of PCRTag amplicons.

b Loss of gene functions is related to the increase of PDV production. Values are averages from three experiments, and error bars denote s.d..

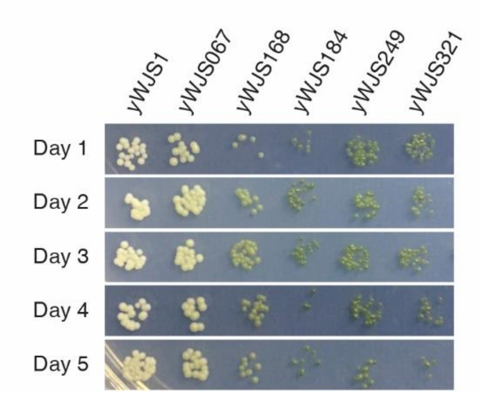

Supplementary Figure 5. Stability analysis of SCRaMbLEants. Phenotypic analysis of the SCRaMbLEd strains on SC–Leu plate at 30ºC for 72 hours for mitotic stability verification after ~60 generations in SC–His medium. SC–Leu, synthetic complete medium lacking leucine; SC–His, synthetic complete medium lacking histidine.

Supplementary Data 1. Segment division, features and PCRTags of ring_synV.

Supplementary Data 2. Survival colonies after SCRaMbLE during Cre induction.

Supplementary Data 3. Strains used in this study.

Supplementary Data 4. Primers used in this study.
