# Supplementary material for: Ring synthetic chromosome V SCRaMbLE
Source: Nat Commun. 2018 Sep 17;9:3783. doi: 10.1038/s41467-018-06216-y (PMC6141504; doi:10.1038/s41467-018-06216-y)
Supplement: Supplementary file 3 — Description of Additional Supplementary Files [file 41467_2018_6216_MOESM3_ESM.docx]

**Description of Additional Supplementary Files**

File Name: Supplementary Data 1

Description: Detailed information about ring_synV chromosome including the segment division, features and PCRTags in every segment.

File Name: Supplementary Data 2

Description: Survival colonies isolated after SCRaMbLE during Cre induction.

File Name: Supplementary Data 3

Description: Strains used in this study.

File Name: Supplementary Data 4

Description: Primers used in this study.
